# Stiffness in vortex—like structures due to chirality-domains within a coupled helical rare-earth superlattice

**DOI:** 10.1038/srep19315

**Published:** 2016-01-13

**Authors:** Amitesh Paul

**Affiliations:** 1 Technische Universität München, Physik Department, Lehrstuhl für Neutronenstreuung, James-Franck-Straße 1, D-85748 Garching b. München, Germany

## Abstract

Vortex domain walls poses chirality or ‘handedness’ which can be exploited to act as memory units by changing their polarity with electric field or driving/manupulating the vortex itself by electric currents in multiferroics. Recently, domain walls formed by one dimensional array of vortex—like structures have been theoretically predicted to exist in disordered rare-earth helical magnets with topological defects. Here, in this report, we have used a combination of two rare-earth metals, *e.g.* 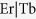 superlattice that leads to long range magnetic order despite their competing anisotropies along the out-of-plane (Er) and in-plane (Tb) directions. Probing the vertically correlated magnetic structures by off-specular polarized neutron scattering we confirm the existence of such magnetic vortex—like domains associated with magnetic helical ordering within the Er layers. The vortex—like structures are predicted to have *opposite chirality*, *side—by—side*, and are fairly unaffected by the introduction of magnetic ordering between the interfacial Tb layers and also with the increase in magnetic field which is a direct consequence of screening of the vorticity in the system due to a helical background. Overall, the stability of these vortices over a wide range of temperatures, fields and interfacial coupling, opens up the opportunity for fundamental chiral spintronics in unconventional systems.

Observing magnetic domains involving exotic magnetic ordering has been a challenge. Particularly interesting are the helical antiferromagnetic rare-earth materials where there is a breakdown of time reversal symmetry as well as space inversion symmetry with important consequences for the structure with topological defects^1^,^2^. In these structures the moments align in ferromagnetic planes within an atomic plane but turn by a characteristic angle along the magnetic propagation vector for the successive atomic plane. Topological defects manifests in vortices and domain walls. Chirality-domains may develop within such systems as the turn angle may have right—handedness or left—handedness sense of spiral. Attempts have been made to image such domains using neutron topography^3^ and also recently by using circularly polarized light^4^. The magnetic superstructure within rare-earths results in the appearance of satellite peaks on either sides of the Bragg diffraction peaks at 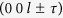. The ± sign designates the handedness of the spiral. Domain walls, developed due to a change in chirality, are predicted to be generically characterized by two-dimensional pattern with regular lattice of vortex singularities, in contrast to more commonly found Bloch or Néel walls^2^,^5^.

In this report, using depth sensitive polarized neutron scattering measurements, we provide quantitative estimations of the magnetic domains within nanometric two-dinesional Er layers. In the bulk form, Er spins are sinusoidally modulated along the c-axis when they are cooled below 84 K. Additionally, the basal plane component orders below 52 K into a helical structure, resulting in a complex magnetic structure. Passing through a number of commensurate propagation vectors, finally a cone structure is developed below 20 K. A schematic of basal plane and conical phase transformation of magnetic moments has been shown in Fig. 1.

In our system, the Er layers are separated by thin Tb layers as they form together a 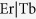 multilayer with competing anisotropy. Such a competition arises, since Tb has its magnetic easy direction in the hexagonal basal plane, while Er has its easy axis along the c-axis. Novel magnetic ordering and novel magnetic phases, originating from these competing anisotropies are not uncommon^6^,^7^,^8^,^9^,^10^,^11^,^12^,^13^,^14^. For bulk Tb, the helical structure exists only between 229–221 K, below which it orders ferromagnetically with an easy axis along the a-axis. Breakdown of translational symmetry in finite helices, due to the presence of interfaces, has substantial influence on their properties^15^. In our multilayer, we could therefore monitor the modulation of the Er helix in presence of a possible ferromagnetic and/or antiferromagnetic coupling between the successive Tb layers with a variation in temperature.

## Results

**Figure 1:**
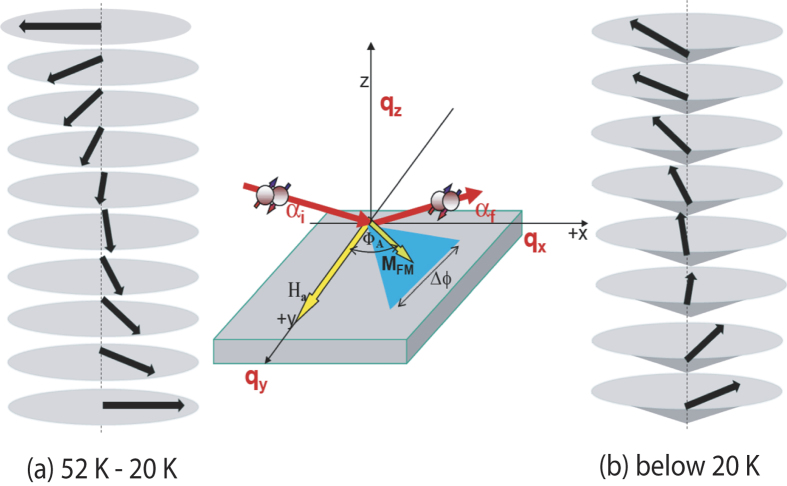
Sketch of (a) basal-plane helix and (b) conical phase with non-zero moment along the out-of-plane axis in Er. The middle inset shows a sketch of the neutron scattering geometry.

In specular scattering geometry (*i.e.*, the angle of incidence 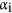 equal to the exit angle 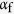), the reflectivities follow from energy and in-plane momentum conservation laws as normal wave vector transfers 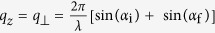 are probed. We consider sample surface in the *x*-*y* plane and the *z*-axis along the surface normal. Off-specular scattering contributions along the in-plane momentum transfer vector 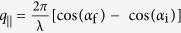 arise, when the in-plane translational symmetry is broken by interface waviness (roughness) or by magnetic domains on a length scale shorter than the in-plane projection of the neutron coherence length 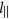 along 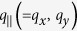
16,17. In the present experimental geometry (see Fig. 1), only 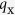 is resolved whereas signal along 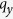 is integrated (collimation along the *y*-axis is relaxed). Since the scattering vector is largely along the surface normal, we are probing the magnetization component in the sample plane coinciding with the *ab* plane of the hexagonal lattice.

Four different cross sections were measured namely, non spin flip (NSF) scattering: 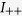 and 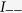, and spin flip (SF) scattering: 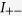 and 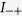. Here + and −signs are used to distinguish the intensity contributions *I* representing a polarization component parallel or anti-parallel to the guiding field, respectively. 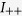 or 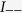 contains the scattering-length densities (SLD) of a magnetic specimen which are given by either the sum or difference of the nuclear 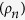 and magnetic 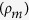 components. Here, 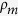 is related to the magnetization 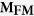. The projection of the longitudinal magnetization onto the neutron polarization axis (y-axis) is proportional to 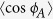, while the projection of the transverse component with respect to the polarization axis onto the x-axis is proportional to 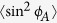. Here, 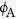 is the angle between the magnetization 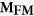 and the applied field H_*a*_, which corresponds usually to the neutron quantization axis. The NSF scattering amplitude provides information about 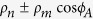, and the SF channels measure 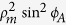. The NSF reflectivities involve squares of the combinations of 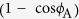 and 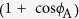 terms while SF reflectivities involve 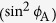 term. Thus, within the one dimensional analysis of the polarization vector (as it is here) it is not possible to discriminate the tilt angle 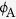 from 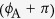
18.

If the domain correlations are smaller than 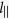, then the neutron wave is scattered in the specular and off-specular directions. If the domain correlations are larger 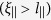, then each domain reflect independently, which are then averaged over all possible domain orientations. Thus, the mean value of the orientation 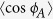 can be obtained. The off-specular scattered intensity thus can be coherent (for periodic structure) or diffuse (for random distribution) with a spatial distribution of 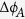. This means that the domain magnetization is randomly tilted by angle 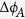 with respect to the mean value averaged over the coherence volume along the transverse 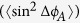 and longitudinal directions 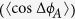, visible in the SF and NSF channels, respectively. Due to the negligible differences between the intensities of the 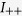 and 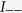 channels, and also between 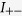 and 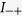 channels, the intensities have been added together in our NSF and SF intensity maps.

Neutron scattering measures the average of the in-plane magnetization vector, which is essentially the incoherent average (averaging intensities) over several coherence volumes (each 5–20 *μ*m in diameter) as defined by in-plane projection of the neutron coherence length 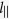. For the neutron reflectometer HADAS, we obtain 
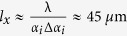
 for 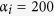 mrad and an uncertainty 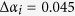 mrad^19^,^20^. Here λ = 0.452 nm is the neutron wavelength. The variation in Δ*α*_*i*_( = S1/L1) can be adjusted by varying the slit opening (S1) before the sample and the distance of the slit from the sample (L1 = 1450 mm).

Earlier, several 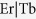 superlattices have been characterized by wide angle neutron diffraction, X-ray resonance exchange scattering and X-ray reflectivity. It was found that long range ordered, modulated magnetic structures exist only in the 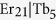 sample below 150 K. An out-of-plane coherence length 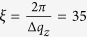 nm or 5 bilayers was deduced from the X-ray data. For the other samples, with different Tb layer thickness (*e.g.* 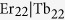 and 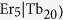, modulated phases without any long range coupling between neighboring layers was observed. The details of which have been given in Refs. 13 and 14. In Ref. 21, the neutron data shown was for the 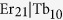 superlattice. Here, we focus on the off-specular neutron scattering measurements from the 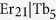 superlattice. The interactions involving 5 monolayers of Tb (which is presented here) is expected to be quiet different, in terms of the ordering of the Er magnetic helix and magneto—elastic interactions involved.

**Figure 2:**
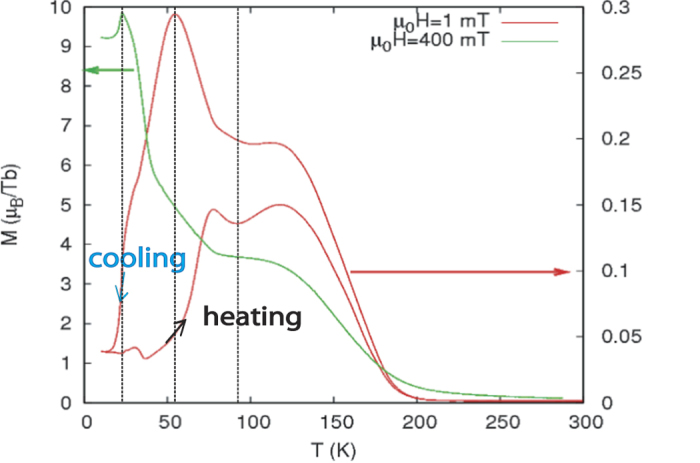
Magnetization versus temperature for two different field cooling options.

Figure 2 shows the longitudinal magnetization M, when a field H ( = 1 mT and 400 mT) is applied within the sample plane. We are primarily sensitive to the Tb moments as they are in the sample plane, while the Er moments, modulating along the c-axis, can have a contribution when they are forming a helix in the basal plane or turning conical or can even cancel out due to domain formation. The measured magnetization is normalized to the number of Tb atoms, determined from the area of the sample and the number of monoatomic Tb layers within the superlattice. Note for comparison, that the saturation moment of Tb is 9.72 *μ*B. The measurements were done upon cooling the sample and subsequent heating at the respective fields. On cooling below T_*C*_ = 230 K, the magnetization (red and green curves) increases steeply indicating ferromagnetic (FM) order of Tb. The curves reach a plateau roughly around the bulk transition temperature of Er 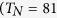 K). This has been attributed to a FM in-plane contribution from the Er moments^22^ in addition to the c-axis modulated structure found by neutron diffraction^14^,^22^,^23^. If the external field is weak (1 mT), the magnetization (red curve) reaches a maximum at 55 K and decreases upon further cooling. In a stronger field (400 mT), the magnetization (green curve) reaches a maximum at 20 K, and then drops. The reduction of the magnetization can be linked to the formation of antiferromagnetic coupling (AFc) of the FM Tb layers^14^. A thermal hysteresis can also be seen comparing the cooling and heating curves measured at 1 mT.

**Figure 3:**
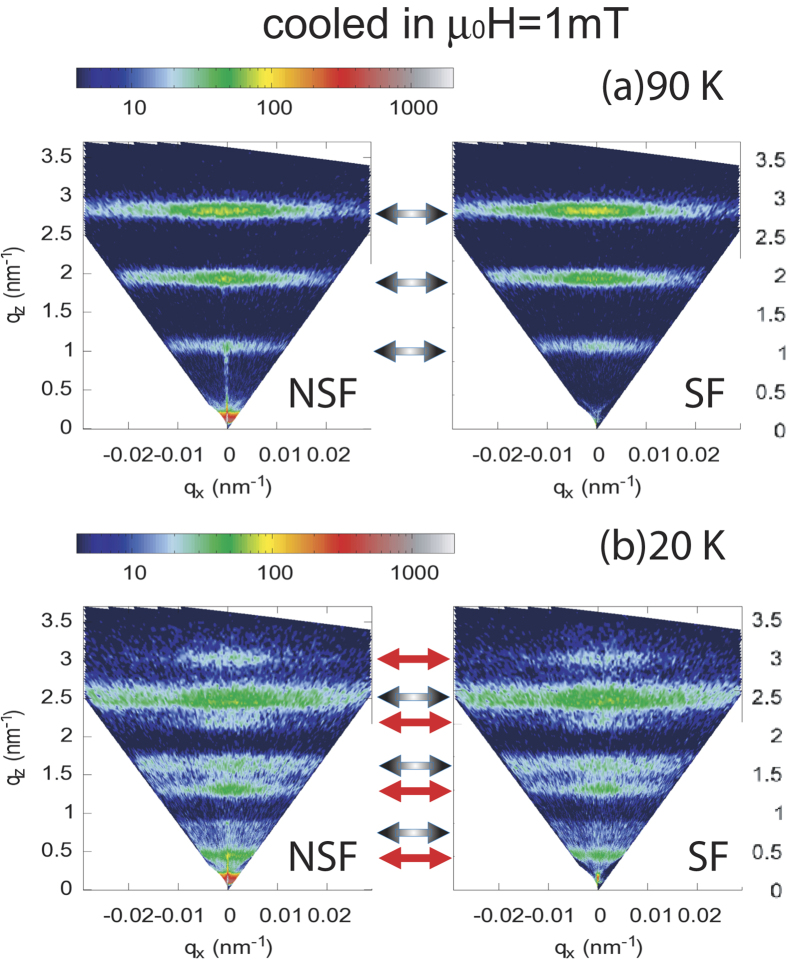
NSF and SF maps of the scattered intensity as function of *q*_x_ and *q*_z_ (in units of 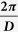) at T = 90 K (**a**) and T = 20 K (**b**), when the sample is cooled in a field of μ*_0_H* = 1 mT. False color code represents the intensities normalized to the monitor counts. The helix—sheets are indicated by the black—grey arrows and the AFc Bragg—sheets by the red arrows.

Next we show the NSF and SF intensity maps for a cooling field 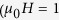 mT) and measured at two different temperatures 90 K and 20 K in Fig. 3(a,b). At 90 K (Fig. 3(a)), we do not observe any superlattice Bragg peak along the specular reflection (at *q*_x_ = 0). This is due to (a) the vanishing SLD contrast between the Er and Tb layers (*ρ*_*n*(*Er*)_ = 7.8 fm and *ρ*_*n*(*Tb*)_ = 7.4 fm) and (b) to the roughness building up through the stack or (c) to variations in bilayer thickness through the 150 repeats which can also smear them out at large q values. The small contribution due to the ferromagnetic order, in a domain state, is not sufficient to increase the contrast between the layers. Instead, we observe off-specular intensity in the form of narrow sheets of coherent intensity indicating long range vertical correlations which can be related to the reminiscent of the helix—like magnetic structure in the bulk with the scattering vectors 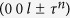:


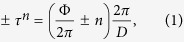


The vector corresponding to these helix—sheets (indicated by the black—grey arrows) is determined by the total turn angle of the helix 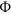. Here, D = 7.3 nm is the bilayer thickness. Thus the structural peak is expected around *q*_*z*_ = 0.86 nm^−1^, which can be seen faintly in the NSF spectra. For a helical magnetic structure, the projection of the ordered moment onto the direction 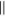 or 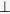 to the neutron polarization axis are the same, rendering similar intensities in the SF and NSF channels. At 200 K (not shown), a complete disappearance of the helix—sheets indicates their temperature dependent magnetic origin.

One may note that the intensity of the helix—sheets shifts from *q*_*z*_ = 2.7 nm^−1^ to 2.5 nm^−1^ (n = 3) when the sample is cooled down from 90 K to 20 K. This indicates a modulation of the helix with temperature. It changes from a helix periodicity D_*helix*_ = 7.0 ± 0.1 nm to around 7.5 ± 0.1 nm. An increase in the lattice constant (along the c-axis) can be linked to the formation of a cone—like structure^22^,^24^,^25^. Interestingly, when the system is cooled down to 20 K, sheets of intensities (AFc—sheets) appear at 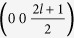 r.sl.u. (as shown in Fig. 3b) at the positions of the half-order Bragg peaks (indicated by the red arrows). Again, there is no specular intensity visible. The intensity sheets are due to the AF domains which are expected due to an AFc ordering between the ferromagnetic Tb moments in the individual layers. The AFc—sheets become stronger with the application of an external field of 400 mT (not shown). The intensities of the half—order sheets in the SF and the NSF channels become comparable, indicating AFc-domains along the six equivalent easy axes of the hexagonal lattice. No significant change in the helix—sheets could be figured out.

**Figure 4:**
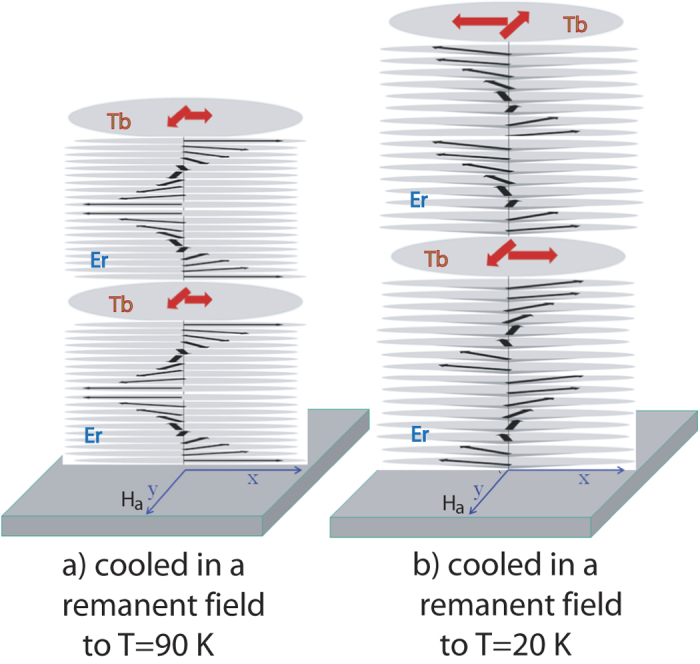
Sketch of (**a**) basal-plane helix of Er and weak ferromagnetic alignment of the domains in the Tb layers at around 90 K and (**b**) conical—helix phase in Er and a predominant antiferromagnetic alignment of the domains in the Tb layers around 20 K after field cooling in *μ*_0_*H* = 1 mT.

A schematic of basal plane and conical phase transformation of magnetic moments for Er and Tb layers has been shown in Fig. 4 representing the situations at 90 K and 20 K, respectively. The sequence of the different phases depends strongly on the composition of the superlattice. For this composition, the basal plane helix of Er is predominant around 90 K^14^. Note that due the insensitivity towards the chirality, NSF and SF signals due to the periodicity of the Er helix remain similar at the two temperatures.

**Figure 5:**
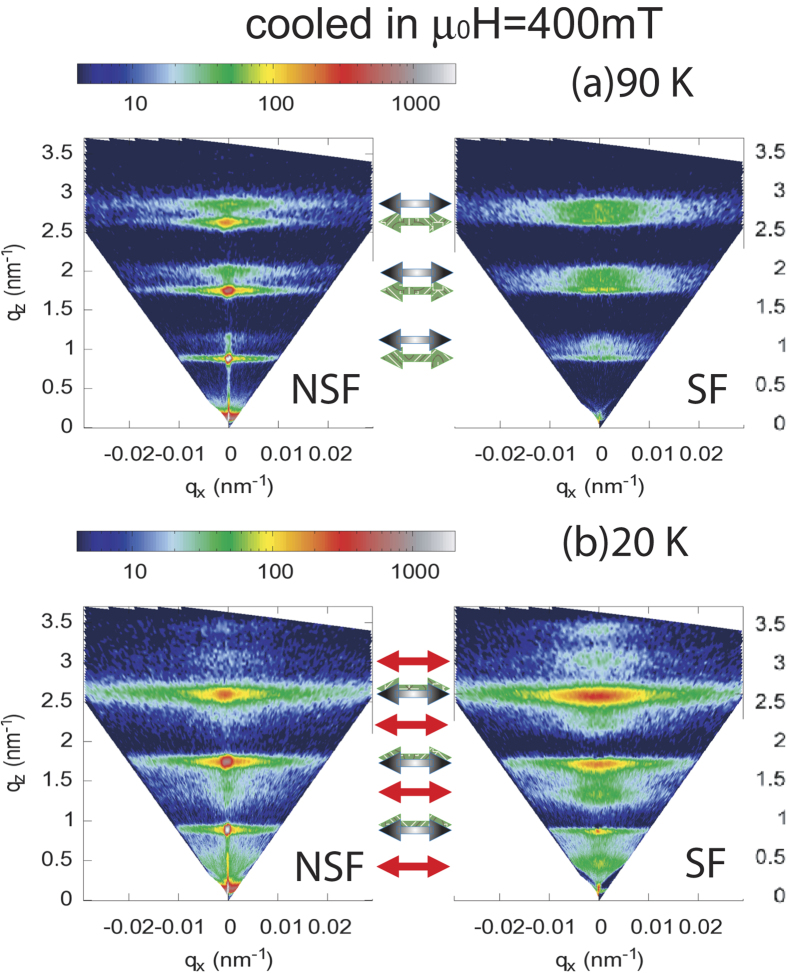
NSF and SF maps of the scattered intensity as function of *q*_x_ and *q*_z_ (in units of 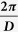) at T = 90 K (a) and T = 20 K (b), when the sample is cooled in a field of *μ*_0_*H* = 400 mT. False color code represents the intensities normalized to the monitor counts. The helix—sheets are indicated by the black—grey arrows, the Bragg—sheets by the green arrows and the AFc Bragg—sheets by the red arrows.

When cooled and measured in a higher field (*μ*_0_*H* = 400 mT), the specular reflectivity (Bragg peaks due to the periodic structure) and the pronounced Bragg—sheets (indicated by the green arrows) at the 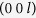 positions appear (see Fig. 5(a)). Ferromagnetic alignments of the Tb moments 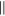 to the field give rise to the specular Bragg peaks at 
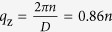
 nm^−1^. Here, unlike the situation of a remanet field cooling 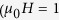 mT), the magnetization is strong enough to render a SLD contrast. The Bragg—sheet intensities are weaker in the SF channel. This signifies that the magnetizations (or the ferromagnetic domains) are predominantly along the applied field axis. One may note that the intensities in the SF channel may also appear due to the leakage of the NSF intensities owing to the inefficiencies of the analyzer (≈88% polarization). The helix—sheets are however still visible, but only at 90 K.

**Figure 6:**
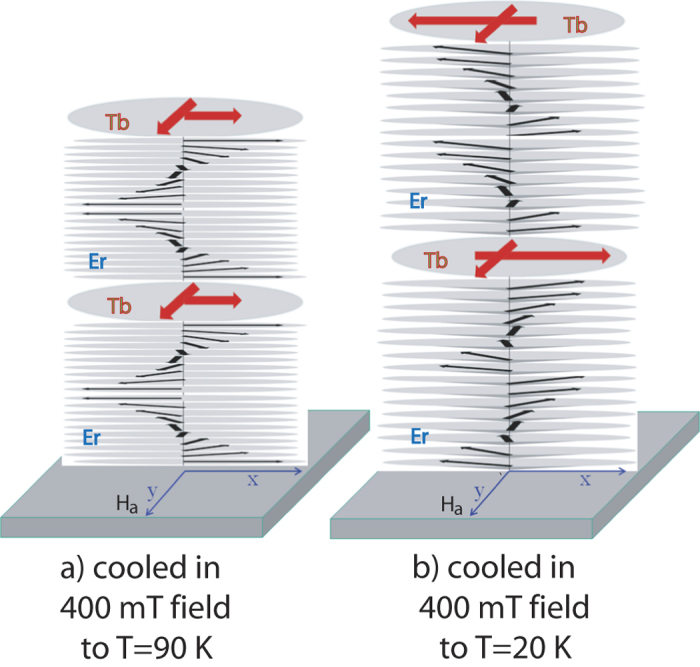
Sketch of (a) basal-plane helix of Er and ferromagnetic alignment of the domains in the Tb layers at around 90 K and (b) conical—helix phase in Er and and a coexistent ferro-antiferromagnetic alignment of the domains in the Tb layers around 20 K after filed cooling in *μ*_0_*H* = 400 mT.

At 20 K (Fig. 5(b)), the helix—sheets intensities remain unchanged (comparing with the measured data in 1 mT) in the NSF and SF channels. In the NSF and SF channels the intensities from the Bragg—sheets and the helix—sheets almost merge together (intensities become stronger and broader). This is a signifies similar periodicities of the helix in Er with the ferromagnetic domains in Tb. Surprisingly, the AFc peaks reappear (note that this is in spite of the large measuring field of 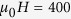 mT) at this temperature. Thus there is a coexistence of FM and AF order within the Tb layers. Interestingly, the AFc—sheet intensities are stronger in the SF channel as compared to the NSF channel. This confirms a stronger antiparallel alignment of the perpendicular component of the sublattice magnetization (easy axis along the x-axis). This is different from the situation when the multilayer was cooled in 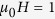 mT. A schematic of basal plane and conical phase transformation of magnetic moments for Er and Tb layers has been shown in Fig. 6 representing the situations at 90 K and 20 K, respectively.

## Discussion

Magnetic order in rare-earth metals results from a subtle balance between the RKKY interaction, anisotropic crystal-field interactions and magneto-elastic interactions. In addition to the RKKY interaction, the interlayer exchange comes into play within a single layer and the epitaxial strains alter the elastic interactions.

It was shown earlier that the helical magnetic phase, found in bulk Tb^25^, is suppressed, if the Tb layer thickness exceeds 10 atomic layers. This suppression can be attributed to a compressive strain, which increases the magneto-elastic energy responsible for the transition from a helical to a ferromagnetic structure (Ref. 9 and references therein). In 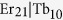 superlattices, for example, the Tb layers order ferromagnetically at 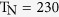 K and couple antiferromagnetically with the next layer of the superlattice^21^. The Er layers show short range incommensurable magnetic structures (IMS) below 100 K. Apparently, different anisotropies of the constituent layers prevent the formation of a long range order for the incommensurable structures. However, in the case of 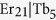 superlattice, the situation is different. One can observe a long range IMS^14^. Below 60 K, the same system shows FM order that is AFc—coupled to the next layer in the stack and this is co-existent with the IMS structure.

Note that Er has undergone a transition from a predominant basal plane helix—type structure to a cone—like structure at 20 K. In bulk Er, a sinusoidal longitudinal phase with an ordering vector 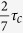, corresponding to a period of approximately 7 layers (here the reciprocal lattice vector along the c-axis is 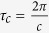) at 84 K, changes to 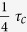 below 52 K. Below 20 K, there is a first order transition to a cone phase which has a ferromagnetic moment along the c-axis and a helical order in the basal-plane component at 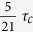
25. It is interesting to note that the turn angle accumulated across 21 atomic layers of Er makes it commensurate with the superlattice periodicity^21^.

Furthermore, it is clear from the neutron data that AF coupling is established for the ferromagnetically aligned Tb moments across the Er layers as we lower the temperature to 20 K. We argue below that pinning the AFc Tb moments across the Er layers can be induced by a periodic chain of vortices within the Er layers with opposite chirality *side—by—side*. The AF order can be plausibly mediated by the commensurate magnetic ordering of the Er moments as it favors pinning of the ferromagnetic Tb domains. These Tb domains induces a change in the chirality of each such vortex which in turn changes the pinned next Tb moment direction. This is also evident from the fact that the long range AFc Tb moments were observed only for the samples with 21 atomic layers. A change in the number of Er monolayers will change the chirality and hence the Tb moment direction.

As long as the condition of translational invariance holds, neutron scattering will not lead to any off-specular scattering. However, when the invariance is broken, off-specular scattering may be seen, which measures the fluctuations around the mean value of the laterally averaged interaction potential. The fact that we observe the off-specular intensities corresponding to the Er helix (helix—sheets), indicates that there is a breakdown of the in-plane translation invariance. Such a breakdown is possible only when lateral domains *with opposite chirality* coexists within a coherence volume. A helical structure alone will not result in off-specular scattering (but only specular scattering) corresponding to the helical periodicity along the film-normal. For a magnetic field perpendicular to the helical axis, the magnetization is expected to remain close to the direction of the field, but as long as the helix is not disturbed (Fig. 5), there will be a formation of vortex walls^26^. The helix—sheets also indicate that the lateral domains are vertically correlated along the z-axis.

The helical ground state in rare-earths originates from an indirect RKKY exchange interaction between local spins in basal planes (nearest-neighbor ferromagnetic (J > 0) and next-nearest-neighbor anti-ferromagnetic (J′ < 0) interaction). These competing interactions are responsible for helical ordering. The local spins thus lower their energy at zero temperature by ordering in a spiral state in which all spins in each plane makes an angle 
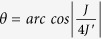
 or the so called helix pitch. Helical magnets demonstrates parity violation in the ground state and a time reversal symmetry breaking allows a continuous phase transformation from helical to magnetic chiral.

In this regard we revisit the model introduced by Nattermann^26^,^27^ for a two dimensional helical magnet. Within the Ginzburg-Landau-Hamiltonian, the density for the localized spins in helical centrosymmetric magnets can be written as


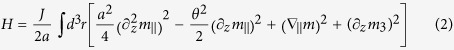


Here, a is the distance between lattice points and 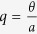 is the magnitude of the helix wavevector where *θ* is the angle between the interplaner spins and 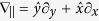. The notation 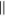 is used to denote the coordinates in the x,y plane. The ground state has a helical structure with


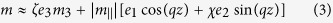


Here, 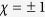 describes the chirality, 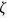 is the conicity of the solution and e are the unit vectors. The out-of-plane component of the magnetization m_3_ can be ignored (thereby ignoring conicity) if we assume m_3_ = 0. Domain walls separate spaces with different values of 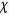 which are important from topological aspect. Here, we can consider a domain wall in the x-y plane, perpendicular to z. The magnetization is periodic along the z-axis with 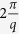 periodicity. Circulating counterclockwise along a closed contour one can observe a change of phase with an integer multiple of 2*π*. Therefore, this contour contains vortices and the vortex energy scales with lattice parameter, unlike the conventional form of Kosterlitz-Thouless vortices that are parallel to the z-axis.

**Figure 7:**
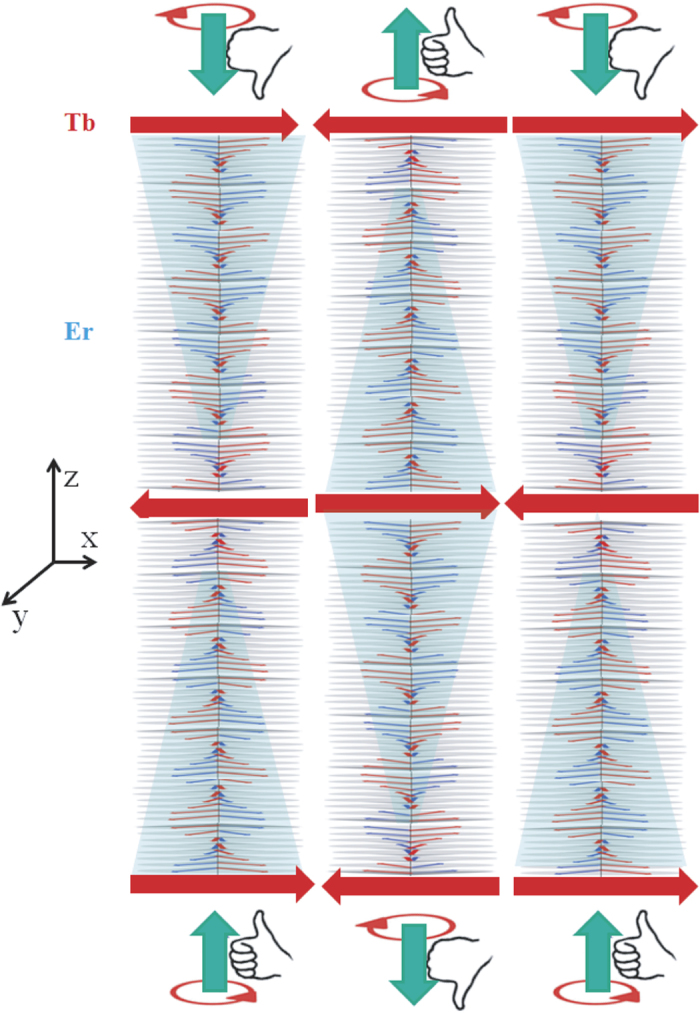
Sketch of the vertically correlated array of chirality-domains due to vortex state in Er and antiferromagnetically coupled domains in the Tb layers across the Er conical—helix at 20 K and in *μ_0_H* = 400 mT.

Vortices are saddle point configurations of the Hamiltonian. The vortices in the domain wall are equidistant with a spacing of 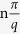. Hubert studied domain walls in helical magnets 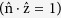 that are free of vortices, perpendicular to the helical axis (z-axis)^28^. The sense of rotation gradually changes from one side of the Hubert wall to the other as one goes along the z-axis. Since neutron scattering is not sensitive to the out-of-plane component of magnetization, we cannot observe the domains separated by Hubert walls. A more general domain wall orientation that makes an angle to the helical axis 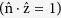 can lead to a *periodic chain of vortices* perpendicular to the helical axis. These vortices can therefore be seen by neutrons as they contribute to the coherent scattering. Domain walls including vortices show strong pinning by impurities. The Tb layers at the Er interface act as pinning centers. Accommodation of a domain wall inside a commensurate region saves energy and this leads to strong pinning for anisotropic systems. The chirality of the vortices defines the pinning direction. Hence the Tb layers, pinned by the Er vortices (with opposite chirality *side*—*by*—*side*), can break up into domains with opposite senses from layer to layer. A schematic of the stacked Er-Tb layer structure with an array of vortex—like structures within the Er layers forming vertically correlated chirality-domains and the AFc-domains of the Tb layers has been shown in Fig. 7, representing the situation at 20 K and in *μ_0_H* = 400 mT.

Images of the chiral domain structure in Ho were obtained earlier by circularly polarized X-ray scanning microscopy imaging^4^. They exhibited a characteristic length scale on the order of 50 *μ*m 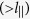, consistent with the previous neutron topography measurements^3^. These dimensions are beyond the accessible length scale of our neutron scattering geometry.

In our multilayer, we extract the widths of the helix—sheets along the 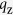 direction by taking vertical line cuts at *q*_x_ = 0 nm^−1^. This yields a vertical correlation length 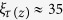 nm for the IMS. This is in agreement with the earlier resonant X-ray scattering results^14^.

Following a similar procedure the lateral (NSF/SF) correlation lengths along the 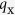 direction (from the horizontal line cuts along 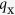 and at a fixed 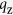 value) are estimated to be around 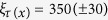 nm and 400(±20) nm when measured at 1 mT and 400 mT, respectively. On the other hand, the lateral correlation length for the vertically coherent AF-domains 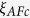 are estimated around 400(±40) nm and 460(±40) nm when measured at 1 mT and 400 mT, respectively. Li *et al.*^2^ indicated that a domain wall between two helix with *opposite chirality*, side by side (their propagation vectors being parallel), must posses a vortex. The estimated lengths can therefore represent the dimension of lateral vortex—like structures plausibly due to the chirality-domains existing within our system.

The inference that the helix—sheets are stemming from some vortex—like structures can be drawn from the following facts: Firstly, we do not see significant differences in the SF and the NSF channels of the intensity maps. This means that the longitudinal and the transverse fluctuations of the moments have equal probability, i.e., either they are at *ϕ* = 45°/45° ± *π* or are distributed regularly in the azimuthal plane. Secondly, the rigidity of the intensities, signifying negligible interaction between the vortex lines in helical structures, can be observed (i) in the presence or in the absence of an applied magnetic field and/or (ii) when an additional magnetic ordering (AF) is introduced within the interfacial Tb layers. It was predicted earlier that the helical background screens the vortex fields over large length scales. This screening perpetuates weak vortex—vortex interaction in the domain wall^5^.

Spins with transverse-spiral (cycloidal) modulation along the specific crystallographic direction, every nearest—neighbour spin pair produces the unidirectional local polarization. The spontaneous polarization can be expressed as.


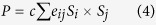


Here, e_*ij*_ is the unit vector connecting the neighboring spins S_*i*_ and S_*j*_, proportional constant c is determined by the spin-orbit and spin exchange interactions as well as the possible spin-lattice coupling term. The polarity depends on spin helicity along the spiral propagation axis. The Dzyaloshinskii-Moriya (DM) coupling gives a preferred chirality to these structures or due to inverse DM, the noncollinear spins produce a nonzero P. The spin helicity can be controlled by the direction of an electric field E^1^. Conceptually, similar to that in magnetic skyrmions^29^, the vortices in 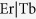 superlattice can also be driven by small currents, which can eventually make them attractive as information carriers. The coupling between current and magnetization, however, may be quiet different here due to the strong intrinsic spin–-orbit coupling in skyrmions.

## Conclusion

In conclusion, we report on the observation of chirality-domains with vortex—like structures in a coupled 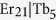 superlattice using polarized neutron scattering technique. Thus far they were predicted to exist only theoretically in disordered helical magnets. On the one hand, the vertically correlated off-specular intensities (forming helix—sheets) corresponds to the helical structure of Er within the rare-earth system at around 90 K. From theoretical models we argue that the vortex—like structures, spanning few hundreds of nanometers in the sample plane, include generically a regular pattern of array of magnetic vortices *side*—*by*—*side*. On the other hand, the Tb moments are seen to form vertically correlated ferromagnetic domains (forming Bragg—sheets) in the presence of an external filed. Furthermore, the ferromagnetic domains within the Tb layers at the Er-Tb interfaces are seen to break up into vertically correlated predominantly antiferromagnetically coupled domains (forming AFc—sheets) below 20 K. This antiferromagnetic coupling is plausibly mediated by the opposite handedness of the individual vortices within the commensurate magnetic ordering of the Er moments (with its transformation from a helical to a conical phase), simultaneously favoring strong pinning of the ferromagnetic Tb domains at each interface. The commensurate ordering of Er with its turn angle plays a crucial role in this case. Stability of the complex helix—sheets over a wide range of temperature, field and interfacial coupling, confirm their characteristic stiffness. Our study thereby provides a significant step towards exploiting the the opportunity for fundamental chiral spintronics.

## Methods

The neutron scattering experiments were performed at the polarized neutron reflectometer with polarization analysis HADAS at the Jülich research reactor FRJ-2 (DIDO). The details of the instrument and scattering geometry has been described in Ref. 19, 20. A position sensitive detector enables the simultaneous record of specular and off specular scattering.

Multilayers with the full layer sequence 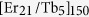 were prepared epitaxially by e-beam evaporation onto 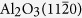 substrates. The indices denote the number of atomic layers. The sample was grown with the hexagonal 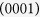 direction parallel to the surface normal, *i.e.* parallel to the propagation vector of magnetization. Details of the sample growth and characterization have been published earlier^14^. For the magnetization measurement a small piece has been cut from the sample used for the neutron scattering experiments. A SQUID magnetometer (MPMS set-up from Quantum Design) has been used to study the temperature dependent magnetization for different applied fields.

## Additional Information

**How to cite this article**: Paul, A. Stiffness in vortex—like structures due to chirality-domains within a coupled helical rare-earth superlattice. *Sci. Rep.* **6**, 19315; doi: 10.1038/srep19315 (2016).
