# Corrigendum: Stiffness in vortex—like structures due to chirality-domains within a coupled helical rare-earth superlattice

**DOI:** 10.1038/srep26727

**Published:** 2016-06-02

**Authors:** Amitesh Paul

Scientific Reports 6: Article number: 19315;10.1038/srep19315 Published online: 01132016; Updated: 06022016

To better reflect the contributions to this study, the author wishes to make the following changes.

In the Acknowledgements section,

“We are thankful to J. Voigt for the sample preparation and MPMS measurements. Thanks are due to U. Rücker for his assistance during the neutron measurements. We are also thankful to P. Böni and Th. Nattermann for useful discussions. This work was supported by the German Research Foundation (DFG) and the Technische Universität München within the Open Access Publishing Funding Programme.”

should read:

“The neutron data presented has been acquired in collaboration with Jörg Voigt, Emmanuel Kentzinger, Ulrich Rücker and Thomas Brückel (JCNS-2, PGI-4: Scattering Methods, Forschungszentrum Jülich GmbH D-52425 Jülich, Germany). T.B. initiated this research project. J.V. prepared the sample and performed the magnetization measurements together with Esther Pfuhl. The neutron measurements were done by A.P., J.V., E.K. and U.R. All of the above have discussed the data. The data processing (like creation of qx, qz intensity maps and provision of the qz and qx cuts) was done by J.V. The model used for the interpretation and analysis of these data was developed by the author A.P., who also wrote the manuscript. We are also thankful to P. Böni and Th. Nattermann for useful discussions. This work was supported by the German Research Foundation (DFG) and the Technische Universität München within the Open Access Publishing Funding Programme.”

Given the following,

1. The low temperature measurements have been performed at 22 K, when the sample does not strictly show the features of the cone phase, but of the ‘tilted helix’ phase, i.e. a modulation of the c-component of the magnetic moment on top of the helical order of the basal plane components (see Fig. 6 of ref. 14). The data presented in the paper are not strictly sensitive to the c-component of the magnetic moment, as the scattering vector is nearly || c and hence the phases cannot be distinguished properly by the present data.
2. The antiferromagnetic coupling of FM Tb layers is found also in other [Er|Tb] superlattices (see refs 14 and 21 of the paper) which requires further investigations to explain. The author would like to state that the model used in the interpretation of the data is not unique, but aims for viable plausibility. Neutron scattering data alone cannot conclude on the existence of chirality domains in vortex like structures.

As such, in the Discussion section,

“A more general domain wall orientation that makes an angle to the helical axis 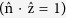 can lead to a *periodic chain of vortices* perpendicular to the helical axis.”

should read:

“A more general domain wall orientation that makes an angle to the helical axis 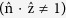 can lead to a *periodic chain of vortices* perpendicular to the helical axis”.

